# An Account of a Case of Amaurosis Cured by Electricity

**Published:** 1788

**Authors:** Miles Partington


V. *An account of a caſe of Amauroſis cured by Electricity. Communicated in a letter to Dr.* Simmons, *by Mr.* Miles Partington.

ANN Mackarel, of Shiffnal, in Shropſhire, aged ten years, had been ſeized, about eleven months before I ſaw her, with a violent pain in her left temple, which was ſoon followed by a remarkable blackneſs of the ſkin of the eyelids of the ſame ſide, extending towards the cheek-bone. To this, in a few days, ſucceeded a dropping of the upper lid over the eye, accompanied with extraordinary ſenſations in the adjacent parts. Her own compariſon of it to 'ſomething galloping' about, may, perhaps, give the beſt idea of the ſymptom.

Application was directly made to an experienced ſurgeon, who gave her nervous medicines, and adviſed her frequently to go into the cold bath. In four or five weeks the lids of her eye returned to the natural ſtate, and ſhe was ſoon after affected with loſs of ſight; the external appearance of the eye remaining without blemiſh.

She continued the uſe of the cold bath ſome time longer, and perſiſted in a long courſe of medicines without any relief. Nine months had now elapſed without any returning viſion, and as ſhe was in good health, with the right eye unimpaired, her parents conſoled themſelves with the hope that no farther misfortune would take place. In this, however, they were deceived, for, on November the 7th, ſhe began to complain of pain and ſenſations, exactly ſimilar to the ſymptoms which firſt attacked her; and her parents in the greateſt diſtreſs came with her to London for farther advice. They carried her to Mr. Pott on Saturday the 15th of laſt November, and and by his recommendation brought her immediately to me to be electrified. It was then I received from them the account I have been giving of the progrefs of the difeafe.

On examining her eyes I could perceive no imperfection in either, except that the left iris remained without motion upon interpofing bodies between it and the light of the window; but I could not fatisfy myfelf that it was præternaturally dilated in any degree. The eye, however, was in fuch a ftate of darknefs that fhe could not difcriminate the window from any other part of the room when the right eye was covered up in which there had, as yet, been no defect; but from the pain and fenfations juft now mentioned, it was feared that the fight of this eye alfo might foon become affected. In every other refpect fhe appeared to be in good health.

I began the operations by placing her in an infulated chair, communicating with a patent electrical machine, by Nairne, the cylinder of which is eleven inches in diameter. The prime conductor was connected with a large Leyden jar, containing four feet of coating, difpofed in the manner I have defcribed in the third edition of of Cavallo's treatiſe on electricity ; and when the jar was fully charged I drew off the contents with a metallic rod, terminating in a wooden point, from the orbits of the eyes, till the jar was exhauſted to three degrees of Henly's electrometer, when the remaining part of the charge was drawn off in ſparks from the left temple and ſuperciliary proceſs. I proceeded much in the ſame manner during the courſe, which conſiſted of thirteen operations, one each day, from Saturday the 15th to Thurſday the 27th of November, and on Monday, December the 1ſt, ſhe went into the country with her ſight perfectly reſtored, and free from every complaint.

The circumſtance which renders this caſe particularly deſerving of attention is, the quickneſs of the recovery; for during the ſecond operation, ſhe perceived an extraordinary glare of light in the room, and on moving my hand before the eye, ſhe could deſcribe the direction of its motion. Upon her going out of my houſe ſhe diſcerned many objects in the ſtreets; and, in the courſe of that day, ſhe had a ſenſation of wearineſs, with great pains in both legs, which was immediately attended with perfect viſion. She came to me the third morning with the ſight as clear as at any part part of her life, and never had the ſmalleſt interruption to this fortunate event to the day of her departure.

*Charles-Street, Middleſex Hoſpital,*

December 6, 1788.

